# Successful Treatment of Actinic Keratosis with Kanuka Honey

**DOI:** 10.1155/2018/4628971

**Published:** 2018-05-31

**Authors:** Saras Mane, Joseph Singer, Andrew Corin, Alex Semprini

**Affiliations:** ^1^Medical Research Institute of New Zealand, Wellington, New Zealand; ^2^Clinical Horizons New Zealand, Tauranga, New Zealand; ^3^Victoria University of Wellington, Wellington, New Zealand

## Abstract

Actinic keratoses form as rough, scaly plaques on sun-exposed areas; they can be an important step in premalignant progression to squamous cell cancer of the skin. Currently, pharmacological treatments consist of topical immunomodulatory agents with poor side effect profiles. Use of honey has been common in both ancient and modern medicine, where it is now a key therapy in the management of wound healing. In vitro studies show the New Zealand native Kanuka honey to have immunomodulatory and antimitotic effects, with recent evidence suggesting efficacy of topical application in a variety of dermatological contexts, including rosacea and psoriasis. Here, we present a case report of a 66-year-old gentleman with an actinic keratosis on his hand, which had been present for years. Regular application of Kanuka honey over three months resulted in remission immediately following the treatment period with no signs of recurrence at nine months.

## 1. Introduction

Actinic keratoses (AK) are common skin lesions that form as rough, scaly plaques of slow growing epidermal keratinocyte dysplasia. They present largely in the elderly as a result of chronic and cumulative sun exposure. Aside from their unappealing cosmetic appearance and irksome tendency to catch on clothing, they can be an important early step in premalignant progression towards squamous cell carcinoma (SCC). As most SCCs arise from AK, it is important that they be recognised and treated early [1].

AK are principally found in sun-exposed areas such as the shoulders, face, hands, ears, and scalp. Incidence varies globally, with 40–50% of Caucasian Australians developing an AK by the age of 40, and around 10% of Caucasian Europeans are reported to be affected [2].

Current treatments tend to favour surgical removal for single lesions or topical immunomodulatory agents such as 5-fluorouracil or imiquimod for those that are contiguous or diffuse [2]. Efficacy of these topical regimes is good when adhered to; however, there are often unpleasant adverse effects including contact dermatitis, burning, or irritation and even systemic flu-like symptoms, which, combined with long duration of treatment, lead to poor compliance. Furthermore, there is no official consensus with regard to guidelines of management, and most guidelines currently published lack a strong evidence base [3].

Use of Manuka and other honeys in the management of wound healing is well established, and recent literature reveals that honey possesses, in addition to antimicrobial action, complex anti-inflammatory and immunomodulatory properties [4]. Subsequently, honey is emerging as an efficacious treatment of other dermatological conditions.

Here, we present a case of resolution of AK by regular topical application of the New Zealand native Kanuka honey.

## 2. Case Report

**Figure 1:**
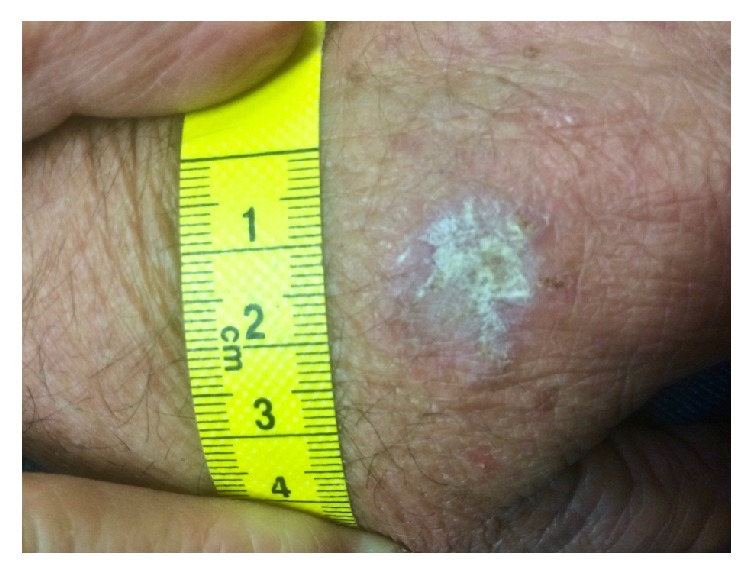
Actinic keratosis on the hand prior to honey treatment.

A 66-year-old Caucasian gentleman presented to his GP with a singular, raised, crusted, scaly lesion of 21 × 20mm size with marginal erythema on the dorsum of his left hand (Figure 1). He reported that the lesion was present for several years but had noted recent growth.

Medical history included AK, basal cell carcinoma (BCC), and seborrheic keratoses in various distributions over recent years, putting him at a higher risk of keratinocyte carcinoma [5]. The lesion was diagnosed in the primary care setting as an AK, though possibility of BCC and SCC was considered.

The previous BCC had been managed successfully with six weeks of topical imiquimod treatment. Procedural removal of the AK was offered to the patient, but he expressed interest in trying a different approach. The patient was contemporaneously enrolled in a clinical trial examining the use of Kanuka honey on rosacea [6] and decided to try using the Kanuka honey topically on his AK.

Honevo® medical grade Kanuka honey (90% Kanuka honey, 10% glycerin) was topically applied once daily using a small amount on the fingertip rubbed into the lesion and surrounding 5 mm of normal skin for 10–30 seconds. It was left on for 30–60 minutes and then washed off with water. This was done consecutively for five days, after which the patient took a treatment break of two days due to lesion tenderness. During the break, the lesion was gently picked at, thereby debriding it. This process was repeated for a total of three months; there were no other treatments used prior to or during this regimen and there were no adverse reactions. The lesion gradually reduced in size with an initial rapid reduction in its dry, crusted nature.

**Figure 2:**
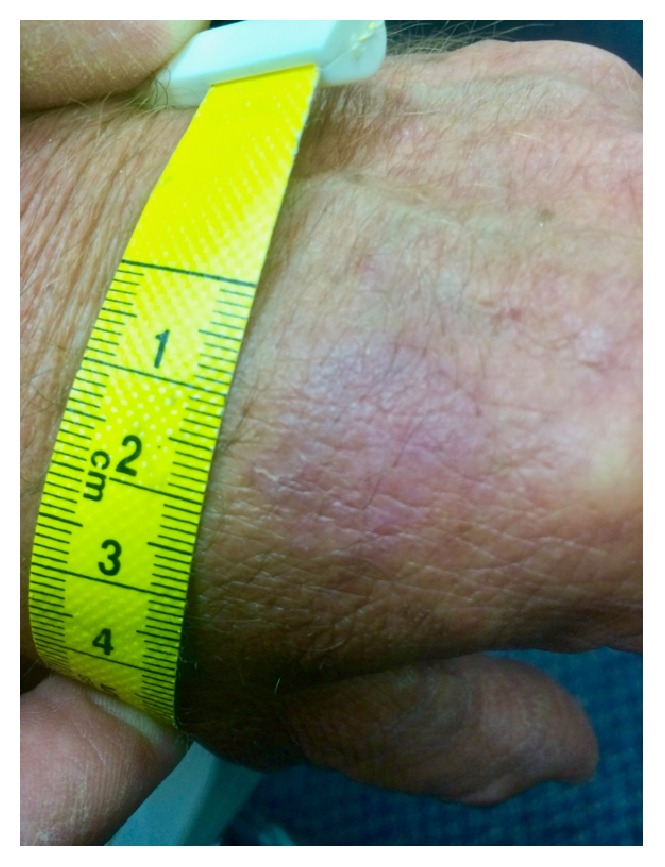
Site of actinic keratosis at three months (immediately after honey treatment).

**Figure 3:**
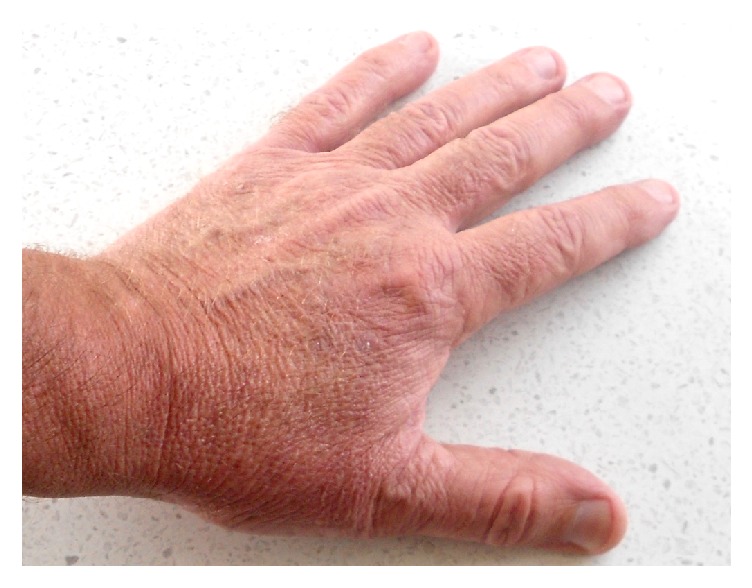
Hand at two years after treatment, showing normal skin and no signs of recurrence.

After three months, residual appearance of the lesion was a 20 mm by 17 mm area of pink skin with no elements of hypertrophy, crusting, or loss of skin integrity (Figure 2). At six months, there were no signs of recurrence. At nine months, the appearance of the skin had fully returned to normal. A telephone follow-up was conducted at two years after treatment, and the patient reported that his skin in the area was still completely normal and that there were no signs of recurrence. A photograph was taken at this time (Figure 3).

## 3. Discussion

This case is noteworthy, as, with topical application of Kanuka honey, there was remission of a growing AK within three months and skin returned to normal within nine months. A limitation of our case is that we were not able to obtain biopsies of the lesion. The AK was diagnosed and treated in primary care, where it is not usual for AKs to be biopsied, and the decision to write up the case was made after the course of treatment had finished.

The use of honey to treat AK has not yet been documented; a MEDLINE/PubMed and google scholar search for* “honey” AND “actinic keratosis” OR “solar keratosis”* (October 2017) did not find any similar cases in the literature. Although spontaneous resolution of AK is seen in its natural history, the mean time for this is 17 months, with a 15% recurrence rate [7]. This suggests that the properties of Kanuka honey may aid and expedite clearance of AK.

The use of honey has been common in both ancient and modern medicine; it is now well established in wound management, where its antimicrobial properties have been shown to inhibit pathogen growth and facilitate healing [8]. In vitro studies show that honeys exert complex anti-inflammatory and immunomodulatory effects, including stimulation and inhibition of various cytokines from granulocytes, as well as the modulation of production of reactive oxygen species from neutrophils [4].

The immunomodulatory properties of Kanuka honey in particular are thought to be more potent than other New Zealand honeys due to the relatively high concentrations of arabinogalactan proteins present [9]. These proteins have been shown to stimulate release of TNF-*α* from monocytic cell lines in vitro. Immunomodulatory topical agents are already widely used in the treatment of AK as an immune component is evident in its aetiology; immunocompromised patients have 250 times the risk of developing an AK than the general population [2].

In vitro studies are also starting to reveal the significant antimitotic and antiproliferative action of honey on cancer cell lines, including those of breast cancer and colorectal cancer lineages [10]. With relevance to skin cancer, Tualang honey has been shown to decrease proliferation and induce apoptosis of squamous cancer cell lines [11]. Acacia honey has also been shown to pause cell cycle progression of melanoma cell lines in a time- and dose-dependent manner. This is thought to be due to the presence of chrysin, an established antitumour agent, in the honey [12].

Due to its readily topical nature, honey has the potential to be used in a variety of dermatological contexts. A recent pilot randomised control trial demonstrated that topical Kanuka honey decreases objective morbidity of psoriasis lesions compared to traditional aqueous cream, a currently recommended topical agent [13]. Braithwaite et al. conducted a larger, randomised, blinded trial of 138 patients with rosacea (an inflammatory, chronic condition affecting the face), examining the use of topical Kanuka honey. They found that Kanuka was well tolerated and effective in significantly reducing rosacea severity as assessed by a blinded clinical examination [6]. Other reports show success with use of honey in dermatitis and pityriasis [14]. Subsequently, a large trial is now underway, comparing the use of topical aciclovir to Kanuka honey for treatment of active cold sores [15].

In conclusion, when determining treatment of choice for AK, type of lesion, patient preference, price, and availability and tolerance for adverse effects all need to be taken into consideration. Current pharmacological treatments can be effective; however, they often come with undesired side effects including contact dermatitis, burning, or irritation and even systemic flu-like symptoms. The New Zealand native Kanuka honey has been shown to have immunomodulatory and anti-inflammatory effects in vitro and is emerging as a viable and well-tolerated treatment for dermatological lesions. There is, however, a notable paucity of blinded randomised controlled trials regarding honey's use on premalignant dermatological lesions. We hope that case reports such as ours engender further study into this area.

## Data Availability

Data from this manuscript were derived from medical consultations with the patient in the primary care setting, as well as the corresponding medical notes and a series of photographs. The photographs are included as figures within this manuscript and a summary of the consultations and notes is included within the section “Case Report.”

## References

[1] Stockfleth E., Peris K., Guillen C. (2015). A consensus approach to improving patient adherence and persistence with topical treatment for actinic keratosis. *International Journal of Dermatology*.

[2] Costa C., Scalvenzi M., Ayala F., Fabbrocini G., Monfrecola G. (2015). How to treat actinic keratosis? An update. *Journal of Dermatological Case Reports*.

[3] Kirby J. S., Scharnitz T., Seiverling E. V., Ahrns H., Ferguson S. (2015). Actinic Keratosis Clinical Practice Guidelines: An Appraisal of Quality. *Dermatology Research and Practice*.

[4] Majtan J. (2014). Honey: An immunomodulator in wound healing. *Wound Repair and Regeneration*.

[5] Schmitt J. V., Miot H. A. (2012). Actinic keratosis: A clinical and epidemiological revision. *Anais Brasileiros de Dermatologia*.

[6] Braithwaite I., Hunt A., Riley J. (2015). Randomised controlled trial of topical kanuka honey for the treatment of rosacea. *BMJ Open*.

[7] Werner R. N., Sammain A., Erdmann R., Hartmann V., Stockfleth E., Nast A. (2013). The natural history of actinic keratosis: a systematic review. *British Journal of Dermatology*.

[8] Carter D. A., Blair S. E., Cokcetin N. N. (2016). Therapeutic Manuka Honey: No Longer So Alternative. *Frontiers in Microbiology*.

[9] Gannabathula S., Skinner M. A., Rosendale D. (2012). Arabinogalactan proteins contribute to the immunostimulatory properties of New Zealand honeys. *Immunopharmacology and Immunotoxicology*.

[10] Erejuwa O. O., Sulaiman S. A., Ab Wahab M. S. (2014). Effects of honey and its mechanisms of action on the development and progression of cancer. *Molecules*.

[11] Ghashm A. A., Othman N. H., Khattak M. N., Ismail N. M., Saini R. (2010). Antiproliferative effect of Tualang honey on oral squamous cell carcinoma and osteosarcoma cell lines. *BMC Complementary and Alternative Medicine*.

[12] Pichichero E., Cicconi R., Mattei M., Muzi M. G., Canini A. (2010). Acacia honey and chrysin reduce proliferation of melanoma cells through alterations in cell cycle progression. *International Journal of Oncology*.

[13] Fingleton J., Sheahan D., Corin A., Weatherall M., Beasley R. (2014). A randomised controlled trial of topical Kanuka honey for the treatment of psoriasis. *Journal of the Royal Society of Medicine*.

[14] Burlando B., Cornara L. (2013). Honey in dermatology and skin care: A review. *Journal of Cosmetic Dermatology*.

[15] Semprini A., Braithwaite I., Beasley R. *5% Aciclovir or Honevo(tm) as a treatment for cold sores*.

